# Supplementary material for: The Immunological Epigenetic Landscape of the Human Life Trajectory
Source: Biomedicines. 2022 Nov 11;10(11):2894. doi: 10.3390/biomedicines10112894 (PMC9687906; doi:10.3390/biomedicines10112894)
Supplement: Supplementary file 1 [file biomedicines-10-02894-s001.zip › biomedicines-1986193-supplementary.pdf]

Gene Name	Forward primer	Reverse primer	Probe*
*HLA-DQA1*01*	GAAGGAGACTGCCTGGCG	CATGATGTTCAAGTTGTGTTTTGC	CCTGCGGGTCAAAACCTCCAAATTTG
*HRM: HLA-DQA1*01*	TATGAGGTTAGGAGTTTAAGATTAG	AAAATCCCCTATAATAACATCTCAATTAC	
*HLA-DQB1*06*	CTTCGTCTCAGTTATGTCTTGGAAG	GAGTCTCTGCCCTCAGCCTGTA	CTTCGGGTAGCAACTGTCACCTTGATGCT
TINA	CCTGAGAGCTGTCTGGATGT	CACTGTGCTCACAGAGTACC	AGCCTGGCACTCCCAGGTGGT
PPIA	Assay id: Hs04194521_s1	Cat. # 4331182	Supplier: Thermo Fisher Scientific
PARTICLE	Assay id: Hs03847241_s1	Cat. # 4426961	Supplier: Thermo Fisher Scientific

Table S1: Sequences of primers and probes (5’ – 3’) used in this study. Reporter and quenchers were 5’FAM – 3’TAMRA respectively for probes except for listed assays which were 5’FAM – 3’NFQ - MGB.
